# Investigation of the effect of chitosan and silver nanoparticles on the antibiotic resistance of *Escherichia coli*O157:H7 isolated from some milk products and diarrheal patients in Sohag city, Egypt

**DOI:** 10.14202/vetworld.2020.1647-1653

**Published:** 2020-08-20

**Authors:** Alshimaa A. Hassanien, Eman M. Shaker

**Affiliations:** 1 Department of Zoonoses, Faculty of Veterinary Medicine, Sohag University, Sohag 82511, Egypt; 2 Department of Milk Hygiene, Faculty of Veterinary Medicine, Sohag University, Sohag 82511, Egypt

**Keywords:** chitosan nanoparticles, diarrheal patients, *Escherichia coli* O157:H7, kariesh cheese, labena, silver nanoparticles

## Abstract

**Background and Aim:**

Antimicrobial-resistant *Escherichia coli* O157:H7 causes serious diseases in humans, especially when circulated in their food. This study was designed to detect the presence of *E. coli* O157:H7 using the *fliC* H7 gene in some milk products as kareish cheese, labena, and yoghurt sold in Sohag city, Egypt, and among diarrheal patients admitted to governmental hospitals in Sohag and also to highlight the risk factors associated with their infection. In addition, the antimicrobial resistance and the effect of chitosan nanoparticles (CNP) and silver nanoparticles (SNP) on *E. coli* O157:H7 isolates obtained from both milk products and patients were investigated.

**Materials and Methods:**

Microbiological culture methods and polymerase chain reaction were used for detecting *E. coli* O157:H7 in 150 milk products and 150 stool samples. Resistance against some antimicrobials that were used in the treatment of animals and humans was investigated using the disk diffusion technique. CNP and SNP at two concentrations (30 and 60 μg/mL) and average sizes of 25.1 and 26.5 nm, respectively, were identified by transmission electron microscopy. Their effect on *E. coli* O157:H7 isolates was examined using the well diffusion method. Risk factors for infection were investigated using statistical analysis.

**Results:**

There were 11.3% and 14.7% of milk products and stool samples positive for *E. coli* O157:H7, respectively. These isolates exhibited high antimicrobial resistance to ampicillin, tetracycline, and gentamycin. CNP and SNP demonstrated inhibitory effects on *E. coli* O157:H7 growth, which significantly increased at high concentrations (60 μg/mL), with mean inhibition zones of 31.941±3.749 and 30.681±3.871 mm for CNP in milk products and patient isolates, respectively. The respective values for SNP were 33.588±3.675 mm and 32.500±2.444 mm, indicating a higher bactericidal effect than that of CNP. Regarding risk factors for infection, both young and elderly subjects and those in contact with infected persons and/or having chronic diseases were infected.

**Conclusion:**

CNP and SNP are suitable for both medical and agricultural applications for disease control and enhancement of food quality.

## Introduction

*Escherichia coli* O157:H7 is considered as a serotype of Shiga toxin-producing *E. coli* that is transmitted through the fecal-oral route, primarily through food, especially of animal origin, in the form of undercooked meat and raw milk, and secondarily from person to person. It causes gastrointestinal sickness ranging from watery diarrhea and abdominal cramps to hemorrhagic colitis, hemolytic uremic syndrome, and renal failure. Complications and deaths occur primarily in children and patients with a compromised immune system, especially old age patients [1].

It is known that haphazard use of antimicrobials in human treatment, poultry industry, and livestock as growth promoters results in multidrug-resistant microbial strains, which raises health risks globally, especially in developing countries [2].

In Egypt, several studies have focused on some nanomaterials that possess antimicrobial effects against several pathogenic microorganisms and are used in health care, food safety and preservation, protection of crops, and water treatment. These nanomaterials are also used in several industries such as food packaging materials, cosmetics, and clothing [3].

Chitosan nanoparticles (CNP) are used in the field of biomedicine due to their specific properties such as non-toxicity, biodegradability, and compatibility. They are used in drug delivery systems as a carrier and as antifungal and antibacterial agents due to their interaction with bacterial surfaces that cause intracellular constituent leakage and death of the cell [4,5].

Silver nanoparticles (SNP) bind to the bacterial cell wall surface, which has a negative charge and cause stability interruption and permeability alteration in the envelope of the cell wall and the flow of silver ions inside the cell. It has been reported that interaction of nanoparticles with cell protein and DNA results in interference of cell division and cell death [6].

This study was designed to detect the presence of *E. coli* O157:H7 using the *fliC* H7 gene in some milk products as kareish cheese, labena, and yoghurt sold in Sohag city, Egypt, and among diarrheal patients admitted to governmental hospitals in Sohag and also to highlight the risk factors associated with their infection. In addition, the antimicrobial resistance and the effect of CNP and SNP on *E. coli* O157:H7 isolates obtained from both milk products and patients were investigated.

## Materials and Methods

### Ethical approval and informed consents

This study was done based on the ethical standards of Faculty of Veterinary Medicine, Sohag University, Egypt. Data and samples from patients were collected after informed consent.

### Samples and data collection

From October 2018 to August 2019, 150 milk products were collected from groceries, small markets, and supermarkets, and if possible from all localities in Sohag city, Egypt, such as kareish cheese (50), labena (50), and yoghurt (50). In addition, 150 stool samples were collected from diarrheal patients in two government hospitals in Sohag city. Patient data, including their clinical characteristics, exposure to infection, and medical history, were collected using clinical investigation forms. Samples of milk products and patients’ stool were collected in sterile cups and transported immediately to the laboratory for the bacteriological examination of *E. coli* O157:H7. Milk products were prepared, as described by Sancak *et al*. [7]. It is difficult to examine the same food consumed by patients because clinical symptoms do not appear immediately, and thus, patients cannot exactly identify the food that resulted in their infection, and moreover, the majority of them do not keep any leftovers.

### Isolation and identification

*E. coli* O157:H7 was isolated using MacConkey Sorbitol Agar (HiMedia, India), as described by Sanderson *et al*. [8]. Biochemical identification was done based on APHA [9] and confirmed by polymerase chain reaction (PCR) in the Molecular Biology Unit, Animal Health Research Institute, El-Giza, Egypt (EGAC /ISO/ 17025/2017).

### PCR technique

DNA was extracted from suspected isolates of milk products and diarrheal patients’ stool samples using QIAamp DNA Mini Kit (Qiagen, Germany) according to the manufacturer’s instructions. PCR was performed using the thermal cycler from Applied Biosystems (Bio-Rad, USA) for detecting the *fliC* H7 gene specific for *E. coli* O157:H7 using the primer sequence F: GCGCTGTCGAGTTCTATCGAGC and R: CAACGGTGACTTTATCGCCATTCC (Metabion, Germany) of 625 bp, as described by Fratamico *et al*. [10]. The cycling conditions for PCR were as follows: 94°C for 2 min, 35 cycles at 94°C for 30 s, 56°C for 1 min, and 72°C for 30 s followed by a final extension step at 72°C for 10 min. The PCR products were then visualized using a light transilluminator (Biometra, Germany).

### Sensitivity against antimicrobial agents

The disk diffusion technique using Müller-Hinton agar (Oxoid, UK) was applied according to NCCLS [11] for evaluating the efficacy of 10 antimicrobial agents from different groups against *E. coli* O157:H7 isolates obtained from milk products and patients’ stool samples using Oxoid antibiotic disks. The inhibition zone was measured and interpreted based on Clinical Laboratory Standards Institute.

### Effect of CNP and SNP on *E. coli* O157:H7 isolates

CNP were prepared, as described by Abdel-Razek [12]. Chitosan powder (Oxford Lab Chem, India) was dissolved in acetic acid (1%) and then stirred for 60 min. Sodium tripolyphosphate (TTP) was prepared by dissolving 0.05 mg/mL in deionized water. At room temperature, 1 mL of TTP was added drop by drop to 100 mL of chitosan solution under magnetic stirring. The pH value was adjusted to 4.7 using sodium hydroxide. After further stirring for 20 min, the mixture was centrifuged for 20 min at 10,000 rpm. The resulting precipitate was suspended in distilled water and centrifuged for removing residual sodium hydroxide. The CNP were stored at 4°C until use. SNP were prepared, as described by Ranoszek-Soliwoda *et al*. [13] using silver nitrate crystals and sodium citrate obtained from Sigma-Aldrich, USA. The molar ratio of sodium citrate to silver nitrate was 7:1; silver nitrate was heated under reflux to boiling; and sodium citrate was added to the solution. It was further heated for 15 min and then cooled to room temperature. CNP and SNP were used at two concentrations (30 and 60 μg/mL). The size of both CNP and SNP was measured at the Electron Microscopy Unit, Assiut University, using JEOL-JEM-100CX II, USA microscope. The antibacterial effect was investigated using the well diffusion method, as described by Rajeshkumar and Malarkodi [14].

### Statistical analysis

SPSS version 14.0 (IBM Corp., NY, USA) was used to analyze the relationship between patients’ characteristics and their infection with *E. coli* O157:H7. Isolates were represented as mean and standard error for their antimicrobial resistance against CNP and SNP and analyzed by independent t-test for evaluating the effect of CNP and SNP. p<0.05 was considered to be statistically significant.

## Results

### *E. coli* O157:H7 detection and risk factors

**Figure-1:**
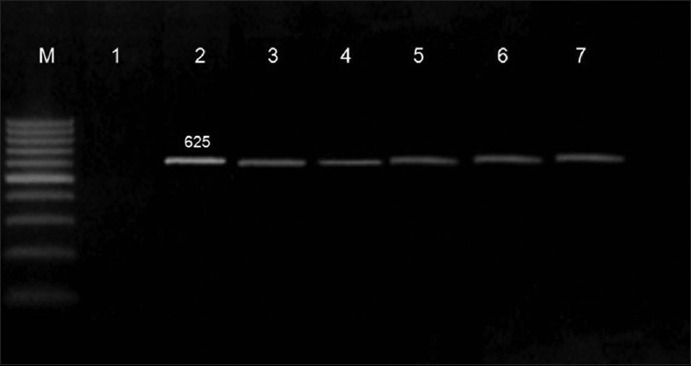
PCR result for E. coli O157:H7 detection. Lane M: 100 bp ladder (Norgen Bioteck), Lane 1: Negative, Lane: 2,3,4,5, 6 and 7: Positive.

Among the 150 milk product samples, 17 (11.3%) had *E. coli* O157:H7 infection, with a higher infection rate in kareish cheese (12 [24%]) followed by labena (3 [6%]) and yoghurt (2 [4%]). Among the 150 patients’ stool samples, there were 22 (14.3%) samples with *E. coli* O157:H7 infection (Table-1 and Figure-1). Patients’ characteristics are shown in Table-2, which indicates an increased infection rate in patients aged <6 years (27.3%) and >55 years (22.7%). Of the 22 infected patients, 18 (81.8%) had chronic diseases such as diabetes mellitus (40.9%), renal disorders (54.5%), hypertension (27.3%), heart disorders (22.7%), liver diseases (13.6%), and bronchial asthma (4.5%). Some patients also had more than 1 disease. Furthermore, *E. coli* O157:H7 infection rate was found to be increased in patients with a history of contact with infected persons and in those with chronic diseases (p<0.05).

**Table-1:** Frequency distribution of *E. coli* O157:H7 in milk products and patients.

Positive samples	Milk products								Diarrheal patients n=150	

	Kariesh cheese n=50		Labena n=50		Yoghurt n=50		Total n=150			
				
	n	%	n	%	n	%	n	%	n	%
*E. coli* O157:H7	12	24	3	6	2	4	17	11.3	22	14.7

n=Number of samples, *E. coli=Escherichia coli*

**Table-2:** Patient characteristics and factors related to infection with* E. coli* O157:H7.

Risk factors	Diarrheal patients n=150		Patients with *E. coli* O157:H7 n=22		p-value
	
	n	%	n	%	
Age^b^					0.815
<6	35	23.3	6	27.3	
6-15	17	11.3	3	13.6	
16-25	14	9.3	1	4.5	
26-35	12	8	2	9.1	
36-45	19	12.7	2	9.1	
46-55	25	16.7	3	13.6	
>55	28	18.7	5	22.7	
Gender^b^					0.936
Female	67	44.7	10	45.5	
Male	83	55.3	12	54.5	
Exposure to infection					
Contact with infected persons^a^	97	64.7	9	40.9	0.05
Contact with animals	14	9.3	3	13.6	0.456
Medical history*^a^	86	57.3	18	81.8	0.05
Liver diseases	24	16	3	13.6	
Renal diseases	31	20.7	12	54.5	
Heart diseases	16	10.7	5	22.7	
Bronchial asthma	39	26	1	4.5	
DM	71	47.3	9	40.9	
Hypertension	63	42	6	27.3	
Pregnancy	16	33.3	1	4.5	

a Significant factors,

b Non-significant factors,

* Some patients suffer from more than one disease, n=Number of samples. *E. coli*=*Escherichia coli*, DM=Diabetes Mellitus

### Antimicrobial resistance

*E. coli* O157:H7 isolates obtained from the majority of milk products and diarrheal patients exhibited multidrug resistance against more than 2 antimicrobials from different groups, with high resistance rates against ampicillin, tetracycline, and gentamycin, but were highly sensitive to ofloxacin and cefoxitin (Table-3).

**Table-3:** Antimicrobial profile of *E. coli* O157:H7 isolated from milk products and diarrheal patients.

Antimicrobial	Milk products isolates n=17						Diarrheal patients isolates n=22					
	
	S		I		R		S		I		R	
					
	n	%	n	%	n	%	n	%	n	%	n	%
Penicillins												
AMP 10 µg	0	0	1	5.9	16	94.1	1	4.5	2	9.1	19	86.4
β-Lactamase												
AMC 30 µg	7	41.2	2	11.8	8	47.1	6	27.3	2	9.1	14	63.6
Cephalosporins												
CRO 30 µg	5	29.4	4	23.5	8	47.1	4	18.2	6	27.3	12	54.5
FOX 30 µg	11	64.7	4	23.5	2	11.8	16	72.7	4	18.2	2	9.1
Aminoglycosides												
CN 10 µg	0	0	3	17.6	14	82.3	3	13.6	3	13.6	16	72.7
AK 30 µg	9	52.9	1	5.9	7	41.2	10	45.5	5	22.7	7	31.8
Tetracyclines												
TE 30 µg	2	11.8	0	0	15	88.2	1	4.5	3	13.6	18	81.8
Quinolones												
CIP 5 µg	7	41.2	5	29.4	5	29.4	10	45.5	6	27.3	6	27.3
OFX 5 µg	15	88.2	2	11.8	0	0	17	77.3	2	9.1	3	13.6
Phenicols												
C 30 µg	1	5.9	5	29.4	11	64.7	10	45.5	4	18.2	8	36.4

AMP=Ampicillin, AMC=Amoxicillin/clavulanic acid, CRO=Ceftriaxone, FOX=Cefoxitin, CN=Gentamycin, AK=Amikacin, TE=Tetracycline, CIP=Ciprofloxacin, OFX=Ofloxacin, C=Chloramphenicol, n=Number of isolates

### Effect of CNP and SNP

As shown in Table-4, CNP exerted an inhibitory effect on the growth of milk product isolates, with mean inhibition zones of 19.705±2.201 and 31.941±3.749 mm at concentrations of 30 and 60 μg/mL, respectively. The mean inhibition zones for SNP were 23.294±3.704 and 33.588±3.675 mm at concentrations of 30 and 60 μg/mL, respectively. Similarly, CNP treatment at concentrations of 30 and 60 μg/mL inhibited the growth of patients’ isolates, with mean inhibition zones of 18.363±2.341 and 30.681±3.871 mm, respectively. However, the mean inhibition zones for SNP were 19.727±3.042 at 30 μg/mL and 32.500±2.444 mm at 60 μg/mL.

**Table-4:** Inhibitory effect of CNP and SNP on *E. coli* O157:H7 isolates from milk products and diarrheal patients.

Concentration (µg/mL)	Inhibition zone (mm)						

	Milk product isolates						

	CNP			SNP			p-value
	
	Min.	Max.	Mean±SE	Min.	Max.	Mean±SE	
30	15	22	19.705±2.201	19	28	23.294±3.704	0.05
60	24	35	31.941±3.749	31	40	33.588±3.675

**Patients isolates**							
	**Min.**	**Max.**	**Mean±SE**	**Min.**	**Max.**	**Mean±SE**	

30	16	22	18.363±2.341	17	27	19.727±3.042	0.05
60	25	36	30.681±3.871	30	39	32.500±2.444

CNP=Chitosan nanoparticles, SNP=Silver nanoparticles, *E. coli=Escherichia coli*, SE: Standard error

## Discussion

The presence of *E. coli* O157:H7 was investigated in some milk products such as kareish cheese, labena, and yoghurt using the *fliC* H7 gene-specific primer, with the detection rates being 24%, 6%, and 4%, respectively (Table-1 and Figure-1). The detection rate in kareish cheese samples was in contrast to the result of similar studies conducted elsewhere in Egypt; for instance, 1.8% and 6.6% as reported by Hussien [15] and El-Kosi [16], respectively. This indicates that other sources of infection may be attributable to the high rates of infection with this organism in Sohag city because kareish cheese is prepared on a small scale in rural homes by traditional methods using raw milk and is sold by street vendors. In fact, raw milk was reported to be a secondary food vehicle for *E. coli* O157:H7 transmission [17]. Although yoghurt and labena are always considered as safe because of their intrinsic nature, they showed a higher percentage of *E. coli* O157:H7 infection than that reported by Hussien [15], which may be due to its tolerance to acidic condition in acidic foods at lower temperature [18]. Similarly, higher infection rates were reported by Chaleshtori *et al*. [19] and Bedasa *et al*. [20] who detected *E. coli* O157:H7 in yoghurt at rates of 10% and 25.7%, respectively. Contamination of yoghurt and labena may be attributed to a number of potential problems that could be identified at diaries, such as inadequate heating of milk or post-pasteurization contamination either by inadequate cleaning of systems or by farm yard matter.

As shown in Table-1, *E. coli* O157:H7 was isolated from 22 (14.3%) diarrheal patients, which is in contrast to the results of Blanco *et al*. [21] and Al-Daragy and Baqer [22] who reported *E. coli* O157:H7 detection rates of 0.5% and 20%, respectively. These variations may be attributed to patients’ immunity, age, medical history, and differences in food culture and food habits, as some people preferred undercooked, rather than well-cooked, meat and favored some milk products such as kareish cheese processed using raw milk rather than sterilized or pasteurized milk. Furthermore, due to the rapid spread of food consumption habits in the community, infection can be transmitted through diseased workers or using infected food materials, inappropriate hygienic measures followed during food handling, and contact with infected persons and animals. Therefore, as the first step in disease prevention, it is important to raise the awareness of people about the disease and its source and mode of transmission and primary hygiene practices.

Regarding patient characteristics shown in Table-2, gender and age showed no correlation with *E. coli* O157:H7 infection, as all age groups had infection at somewhat similar proportions, except children aged <6 years and elders aged >55 years who constituted the majority of *E. coli* O157:H7 infection cases. Contact with infected persons and presence of chronic diseases were significant risk factors for infection (p<0.05). This finding is consistent with that reported by Al-Wgaa and Alwan [23]. However, contact with animals was not found to be a significant risk factor for infection, which in contrast to the finding of Jaros *et al*. [24]; the difference may be related to the patients’ activities and habits. The type of food as a source of infection was not exactly clear; data obtained from patients indicated that a variety of milk and meat products was consumed almost every day that were either prepared at home or fast food. Moreover, patients visited hospitals after 2 or 3 days of illness and the majority of them did not provide accurate data about the consumed food. Therefore, in the present study, milk products as a source of infection were examined for the presence of *E. coli* O157:H7 because patients, especially children, consumed milk products daily.

In Egypt, an indiscriminate approach of antibiotic treatment is represented in the form of haphazard selection of antibiotics without detecting the causative agent of diseases, irregular medication doses, discontinuation of treatment course, and absence of treatment protocols and medication history for either humans or animals. These factors increase the resistance ability of microorganisms against the widely used antimicrobials.

As shown in Table-3, *E. coli* O157:H7 isolates obtained from milk products exhibited resistance to varied groups of antibiotics such as ampicillin (94.1%), tetracycline (88.2%), and gentamycin (82.3%) and susceptibility to ofloxacin (88.2%). The isolates recovered from diarrheal patients also showed high resistance to ampicillin (86.4%), tetracycline (81.8%), gentamycin (72.7%), amoxicillin/clavulanic acid (AMC) (63.6%), and ceftriaxone (54.5%) but were highly sensitive to ofloxacin (77.3%) and cefoxitin (72.7%). These findings may be related to the three resistant antibiotics (ampicillin, tetracycline, and gentamycin) that were widely used in human and animal treatment without previous data regarding medication history and their effects. Similar to our study, Ivbade *et al*. [25] also reported higher resistance rates against ampicillin and AMC (100%) and approximately similar values for tetracycline (88.2%) and chloramphenicol (64.1%). The presence of resistant isolates in milk products emphasizes the transmission of such resistance to humans through food chain, which hinders patients’ recovery and causes prolonged disease duration and death in some cases [26].

### Effect of nanoparticles

The emergence of resistant microorganisms against antimicrobials will, in turn, increase the focus on nanoparticles to explore other effective treatments. Therefore, there has been extensive research focusing on nanotherapeutics that promote drug effectiveness with low toxicity [27]. They may be applied as a polymer in drug delivery methods due to their small size, which facilitates the entry of drugs, especially CNP, into the cell [4]. They can also be used for improving food safety such as in food packaging to increase shelf life and in food preservation without causing any change in physical characteristics and taste of food products [28].

**Figure-2:**
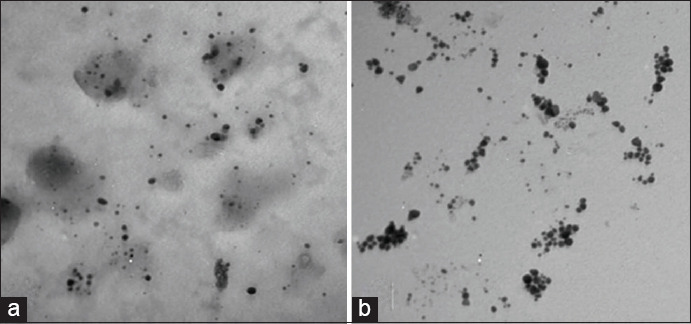
(a) CNP with average size 25.1 nm. (b) SNP with average size 26.5 nm.

In this study, CNP and SNP were used at two concentrations (30 and 60 μg/mL) at average sizes of 25.1 and 26.5 nm (Figure-2), respectively. As presented in Table-4, CNP and SNP exerted a high bactericidal effect on isolates recovered from milk products and patient samples, which was significantly increased with increasing concentrations. This finding may be explained by the fact that Gram-negative microorganisms have a thinner peptidoglycan layer of cell wall than Gram-positive microorganisms, which increases the penetration of nanoparticles into the cell and causes alteration of bacterial DNA [29]. Our results indicated that SNP have a slightly higher effect than CNP, which may be related to the silver ions released inside the cell and the effect on the respiratory chain that interferes with oxygen reduction [30]. Therefore, our results indicate the importance of the application of nanoparticles in biomedicine and food industry to treat diseases and stop pathogenic microbial transmission through food.

## Conclusion

Circulation of *E. coli* O157:H7 isolates that are resistant to the most commonly used antibiotics among patients and milk products necessitates obligatory monitoring of antibiotic use by establishing a therapeutic program for diseases and strict supervision of antibiotic utilization. Moreover, further studies investigating alternative substances to antibiotics such as nanomaterials, probiotics, and natural extracts are required for treating *E. coli* O157:H7 infections.

## Authors’ Contributions

AAH and EMS equally contributed in the idea of the study, designing of the study, collecting data and samples, laboratory work, literature search, data analysis, and wrote and prepared the manuscript. Both authors read and approved the final manuscript.

## Competing Interests

The authors declare that they have no competing interests.

## Publisher’s Note

Veterinary World remains neutral with regard to jurisdictional claims in published institutional affiliation.
